# Supplementary material for: Association between oral microbiome and seven types of cancers in East Asian population: a two-sample Mendelian randomization analysis
Source: Front Mol Biosci. 2023 Nov 21;10:1327893. doi: 10.3389/fmolb.2023.1327893 (PMC10702768; doi:10.3389/fmolb.2023.1327893)
Supplement: Supplementary file 1 [file DataSheet1.ZIP › Supplementary Materials/MR plots for tongue/tongue═╝/Ovarian cancer/pheno.423_to_ovarian cancer_leave_one_out.pdf]

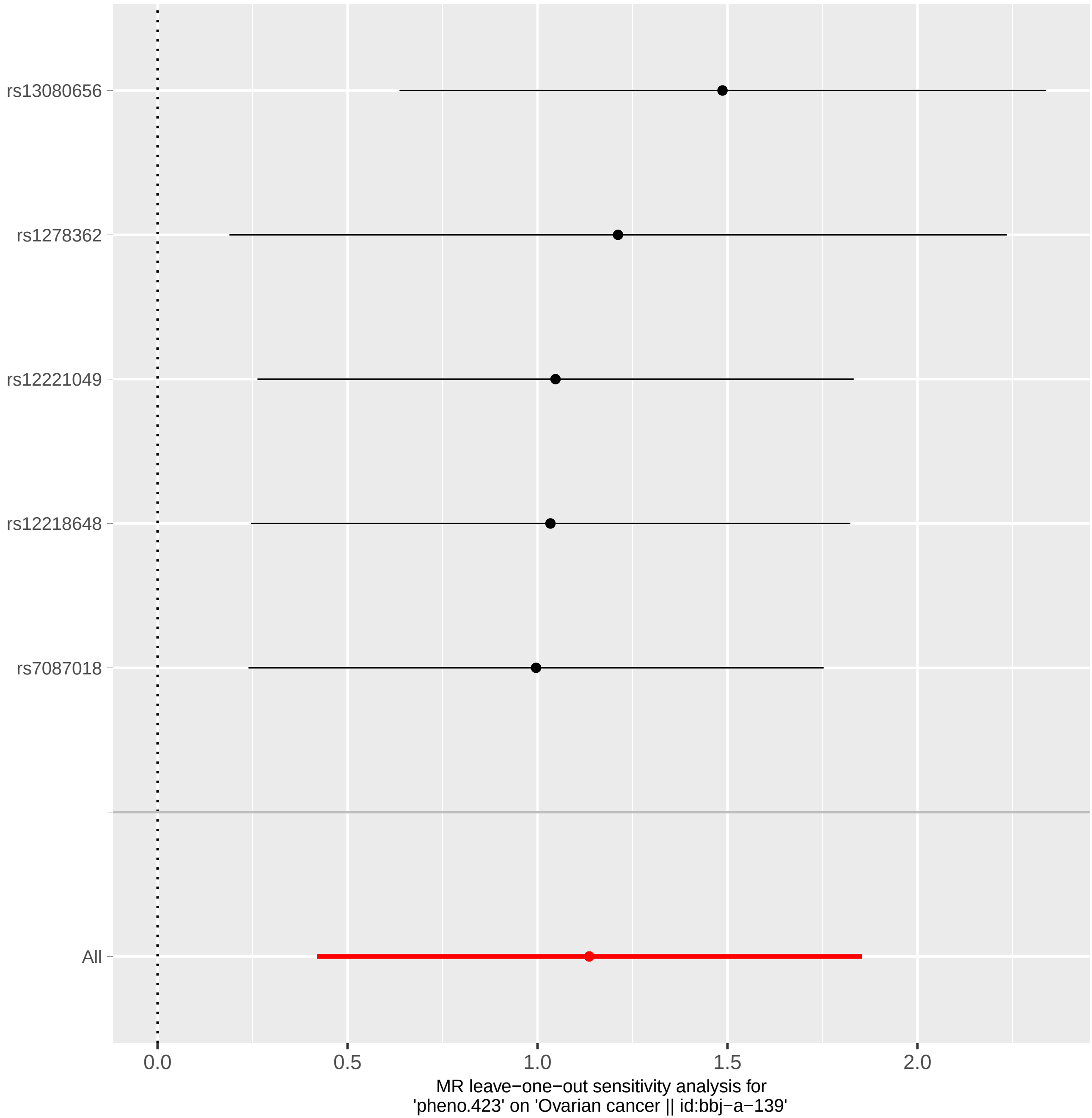
rs13080656
rs1278362
rs12221049
rs12218648
rs7087018
All
0.0
0.5
1.0
1.5
2.0
MR leave−one−out sensitivity analysis for
'pheno.423' on 'Ovarian cancer || id:bbj−a−139'
